# 剂量优化：慢性髓性白血病的个体化治疗策略

**DOI:** 10.3760/cma.j.issn.0253-2727.2022.05.017

**Published:** 2022-05

**Authors:** 怡琳 陈, 菁 邹, 龑莉 张, 纬明 黎

**Affiliations:** 1 华中科技大学同济医学院附属协和医院血液科，武汉 430022 Department of Hematology, Union Hospital, Tongji Medical College, Huazhong University of Science and Technology, Wuhan 430022, China; 2 河南省肿瘤医院、郑州大学附属肿瘤医院血液科，郑州 450008 Department of Hematology, the Affiliated Cancer Hospital of Zhengzhou University, Zhengzhou 450008, China

慢性髓性白血病（CML）是一种（9;22）（q34;q11.2）染色体异常造成的克隆性骨髓增殖性疾病[1]。酪氨酸激酶抑制剂（TKI）的出现显著改善了CML慢性期（CP）的预后，CML-CP患者的年死亡率从2000年的10％～20％下降到1％～2％。CML的患病率逐步上升，并预计在未来几年继续增加，因此CML患者群体将越来越大[2]。目前已有5种TKI（伊马替尼、尼洛替尼、达沙替尼、博苏替尼和普纳替尼）被FDA批准作为CML-CP患者的治疗用药[3]，此外还有在韩国获批上市的拉多替尼和中国自主知识产权的氟马替尼[4]–[5]。使用TKI的患者可以获得与正常人相似的预期寿命[6]，但长期使用TKI伴随较多持续的药物不良事件，影响患者的生活质量，进而影响患者的依从性、治疗疗效[7]–[9]。相比一代TKI，二、三代TKI还会产生一些较为严重甚至有致死可能性的不良事件[10]，如达沙替尼相关的胸腔积液、肺动脉高压，尼洛替尼引起的心血管事件，博苏替尼引起的腹泻和肝功能障碍，普纳替尼引起的高血压、外周动脉闭塞和胰腺功能障碍等。除此之外，长期TKI治疗还会增加患者与社会的经济负担[11]。

目前，愈来愈多的临床研究数据表明，部分获得持续深层分子学反应（DMR）的患者能够实现相对持久的安全停药，即无治疗缓解（TFR）[12]–[14]。虽然停药可解决上述长期服用TKI相关的各种问题，但大约30％的患者停药后出现肌肉骨骼疼痛或关节周围僵硬 [15]。此外，一些患者会因停用TKI而感到严重焦虑，约 50％ 的患者担心失去治疗反应[16]，并且分子学复发的患者在重新开始TKI治疗时经受更为严重的焦虑[17]。更为重要的是，TKI不能治愈CML。自伊马替尼开始应用于CML的治疗至今已有20年，只有5％～10％的患者可以成功停药，仍有80％的患者需要持续治疗以获得长期生存，尽管其中有20％的患者满足停药条件[18]。

考虑到长期服用TKI存在的不良反应、费用等各种问题，以及大多数患者不能成功停药的事实，剂量优化即对患者个体化地减低剂量治疗是否可作为一个折中的策略？剂量优化是否可以在维持疗效的前提下缓解全量用药带来的相关问题，如减少不良反应、提高治疗依从性和减少治疗中断等，此外，剂量优化对TFR有何影响？本文将就相关问题整理综述。

一、剂量优化的理论基础

Fassoni等[19]基于IRIS研究和CML-Ⅳ研究中部分患者的真实数据开发了一个数学模型，将TKI发挥作用的时间过程描述为一个动态过程。CML初始治疗阶段主要由​​TKI对增殖性白血病干细胞（LSC）发挥细胞毒性作用，期间增殖性 LSC 可被迅速清除，BCR-ABL水平迅速降低，而长期的治疗反应则取决于静息LSC的罕见激活。TKI剂量降低至少50％不会导致长期治疗效果的下降，反而能够维持 BCR-ABL水平的继续降低。TKI发挥作用需要TKI血药浓度确保在一定范围内，预测剂量降低下限为原始剂量的25％。该模型认为，对于维持稳定良好分子学反应的CML患者，可考虑将剂量减半作为长期治疗选择，特别是有不良反应和经济压力的患者。

二、剂量优化的临床证据

1. 一线TKI治疗的剂量优化：OPTIM DASATINIB试验是首项一线达沙替尼浓度监测的前瞻性剂量优化研究，旨在探索根据达沙替尼的血浆谷浓度（C_min_）监测（TDM）进行剂量调整的策略。研究纳入287例初始剂量为100 mg/d的患者，根据其服用达沙替尼7～10 d时的C_min_进行分组：浓度较高（C_min_>3 nmol/L）的患者（80例）被随机分配在剂量减少组（TDM组，可根据连续监测的C_min_将剂量以每次20 mg的幅度最低减少至40 mg/d）（38例）和标准组（对照组，100 mg/d）（42例），浓度较低（C_min_≤3 nmol/L）的患者（207例）被分配到观察组（100 mg/d）。研究结果显示：在TDM的初始12个月期间，观察组中位和平均剂量分别为94和100 mg/d，对照组为88和100 mg/d，TDM组为60和55 mg/d。与对照组相比，TDM组胸腔积液的累积发生率显著降低（1、2和3年分别为4％对15％，11％对35％，12％对39％，*P*＝0.0094）。令人欣慰的是，三组患者在治疗12个月和3年时的主要分子学反应（MMR）率差异均无统计学意义。作者认为，虽然不建议所有的患者均以低于100 mg/d的剂量开始治疗，但建议通过药物浓度监测进行剂量优化，可有效减少胸腔积液的发生，并且该研究发现有40％的50岁以上患者血药浓度过高，因此对于这部分患者更应进行TDM，如无条件进行，达沙替尼起始剂量为50～60 mg被认为是可以接受的[20]。

一项非随机性研究评估了低剂量达沙替尼（50 mg/d）对初诊CML-CP患者的疗效和安全性。纳入83例接受达沙替尼50 mg/d一线治疗的CML-CP患者。6个月和12个月的完全细胞遗传学反应（CCyR）率分别为77％和94％。治疗12个月时的累积MMR率、分子学反应4（MR4）率和分子学反应4.5（MR4.5）率分别为81％、55％和49％。21例（25％）患者中断治疗，中断治疗中位时间为13（4～64）d。5例（6％）出现胸腔积液，其中4例（80％）需要减少剂量。这些结果显示50 mg/d达沙替尼治疗初诊CML-CP安全且有效[21]–[22]。值得注意的是，该研究中达沙替尼50 mg/d治疗12个月的CCyR率和MMR率分别为94％和81％，似乎优于DASISION研究中达沙替尼100 mg/d治疗12个月的CCyR率和MMR率（83％、46％）。和100 mg/d相比，达沙替尼50 mg/d相关的胸腔积液和3/4级血液学不良反应显著降低（6％对28％，0对64％）[23]。由于达沙替尼50 mg/d具有良好的疗效和耐受性，2020年版ELN推荐其作为CML-CP患者一线治疗的选择之一[24]。余丹等[24]在一项随机对照研究中对比分析了达沙替尼100 mg/d和70 mg/d治疗CML-CP患者的疗效和安全性。在治疗12个月时，达沙替尼70 mg/d和100 mg/d组的CCyR率分别为87.50％和100％（*P*>0.05），MMR率分别为75％和80％（*P*>0.05），而70 mg/d组不良反应发生率与100 mg/d组相比差异无统计学意义，但有减少趋势[25]。

和年轻患者相比，老年患者服用达沙替尼发生胸腔积液的风险较高[20]。老年患者的达沙替尼剂量优化也是研究者关注的重点。一项研究报告了达沙替尼20 mg极低剂量治疗年龄≥65岁 CML-CP患者的疗效与安全性[26]，研究共纳入21例初诊（16例）、伊马替尼耐药（2例）或不耐受（3例）的患者，患者的中位治疗剂量为26 mg/d，其中91％的患者使用达沙替尼的平均剂量为≤50 mg，72％的患者的平均剂量为≤20 mg。在治疗24个月时，21例患者的MR4和MR4.5获得率分别为62％和29％，达沙替尼剂量为 ≤20 mg的患者的MR4和MR4.5率分别为67％和34％。患者对低剂量达沙替尼治疗的耐受性较好，大多数为1～2级不良反应。29％的患者在6个月内发生胸腔积液，均为1～2级，通过减低剂量和对症治疗而消失。该研究显示老年患者可从达沙替尼剂量≤20 mg治疗中获得理想的分子学反应，且具有较好的耐受性。

2. 二线或二线以上TKI治疗的剂量优化：一项回顾性研究分析了真实世界达沙替尼治疗相关胸腔积液发生情况[27]。研究收集了853例一线或二线接受达沙替尼治疗患者的数据，共196例（23％）发生过不同程度的胸腔积液，其中162例（82.6％）患者为伊马替尼耐药或不耐受患者，38例（17.4％）患者为初诊CML患者。在第一次出现胸腔积液时，33.6％的患者未获得MMR，28.6％处于MMR，37.8％处于DMR。在发生胸腔积液的患者中，有71.9％（141/196）的患者曾暂时中断达沙替尼，59.2％（116/196）的患者降低达沙替尼剂量，有29.1％（57/196）的患者最终停止治疗。值得注意的是，在剂量减低的患者中，59.5％（69/116）的患者中出现胸腔积液的复发。因此，对于已经获得MMR或DMR的患者，在胸腔积液发生前减少剂量可能有助于降低这种特异性不良反应的发生率。

在另一项研究中，52例达沙替尼、尼洛替尼或博苏替尼不耐受的患者接受普纳替尼治疗，大多数患者给予30 mg/d（18例）或15 mg/d（25例）治疗，疗效不佳的患者（9例）给予45 mg/d治疗并根据疗效减低剂量[28]。在普纳替尼中位治疗的19.2个月内，21 例（40.4％）患者的分子学反应得到提高（包括6例MMR和15例DMR患者）。在普纳替尼15 mg/d治疗的患者中，10例（40％）的治疗反应提高至DMR。共有46.2％（24/52）患者出现不良反应，包括胰腺炎（13.5％）、高血压（11.5％）、心血管不良反应（7.7％）和其他不良反应（13.5％）。该研究结果显示：降低剂量的普纳替尼可作为其他TKI不耐受的患者的一种有效的治疗选择。

3. 获得MMR或DMR的患者的剂量优化：在INTERIM研究中，76例年龄≥65岁接受伊马替尼治疗超过2年处于稳定的CCyR和MMR患者参与伊马替尼间歇治疗（400 mg治疗1个月，暂停1个月）。至少随访6年时，76例患者中有16例（21％）丧失了CCyR和MMR，16例（21％）仅丧失了MMR，失去治疗反应的患者恢复伊马替尼连续治疗均重新获得CCyR、MMR或更深层次的分子学反应。间歇治疗期间，50％的患者的不良反应消失，特别是肌肉疼痛和痉挛，以及液体潴留[29]。在另一项伊马替尼减量研究中，43例接受伊马替尼400 mg治疗获得DMR（中位维持时间4.1年）的患者接受伊马替尼300 mg/d治疗[30]，减量前分子学反应为MR4、MR4.5和MR5.0的患者分别有6、28和9例。在末次随访时，只有1例患者失去了DMR，所有患者均维持MMR。分子学反应为MMR、MR4、MR4.5和MR5.0的患者分别有1、3、9和30例。在37例减量前有不良反应的患者中，有23例（62.2％）观察到不良反应改善。该研究提示对标准剂量伊马替尼有持续DMR的CML患者，剂量减少至300 mg/d可显著提高耐受性并保持疗效。另一项研究中，52例至少获得MR4的患者接受低剂量伊马替尼（26例）、尼洛替尼（6例）、达沙替尼（19例）、博苏替尼（1例）治疗，在低剂量中位治疗25个月时，绝大多数患者（50/52）仍维持MR4。在最终随访时，4例患者处于MR4，47例患者处于MR4.5或更深层次的分子学反应，5年预测维持MR4生存率为91％以上，结果显示：低剂量TKI可以有效维持治疗反应并且降低TKI相关的不良事件[31]。

NILO-RED研究是一项观察性、多中心研究，旨在评估标准尼洛替尼每日2次方案治疗转换为每日1次方案的可行性[32]。研究共纳入了一线（46例，300 mg每日2次）和二线尼洛替尼（21例，400 mg每日2次）治疗的患者，剂量调整前分子学反应为MMR、MR4和≥MR4.5的患者分别有9例（13.4％）、17例（25.4％）和41例（61.2％）。调整后的尼洛替尼剂量为450 mg每日1次（86.6％）、400 mg每日1次（10.4％）或300 mg每日1次（3.0％）。在尼洛替尼减量后的末次随访时，分子学反应为MMR、MR4和≥MR4.5的患者分别有4例（6.3％）、11例（16.4％）和52例（77.6％）。研究表明，标准尼洛替尼每日2次方案治疗改用尼洛替尼每日1次维持治疗是可行且安全的。

4. TFR前的剂量优化：TKI减量治疗对获得TFR的影响也是研究者关注的重点。DESTINY研究是一项CML患者经伊马替尼、尼洛替尼或达沙替尼治疗达到稳定MMR以上疗效后，减半用药剂量12个月后再停药的临床试验[33]。该研究纳入174例接受TKI治疗至少3年且获得MMR（49例）或DMR（125例）至少12个月的患者。患者先接受12个月 50％标准剂量的TKI治疗：伊马替尼200 mg每日1次（148例）、尼洛替尼200 mg每日2次（16例）和达沙替尼50 mg每日1次（8例）。在TKI剂量减半的12个月内，DMR组较MMR组有更低的分子学复发率（定义为丧失MMR），分别为2.5％和18.8％（*P*＝0.0007）。所有复发的患者在重新开始全剂量TKI治疗4个月内均重新获得MMR。在减半12个月后，100例患者进入TFR阶段，其中DMR组患者有84例，MMR组患者有16例。DMR组较MMR组可获得更高的2年无复发生存率（72％和36％）。预测影响复发的唯一因素是TKI治疗持续时间。DESTINY研究结果显示，对于大多数获得较好疗效的CML患者来说，降低TKI剂量是安全可行的，并且在停药2年后仍可维持较好疗效。对于只获得MMR的患者，减量后停药的TFR率显著低于获得DMR的患者，这类患者的减量可能会延迟或阻止TFR的实现。因此，对于有停药需求的患者，仅达到MMR时的减量需要谨慎对待。在更新的DESTINY试验研究中，174例患者在TKI剂量减半期间对BCR-ABL^IS^值进行监测[34]。结果显示：剂量减半期间根据log（BCR-ABL^IS^）量表测定的斜率参数每月额外增加0.01 log（BCR-ABL^IS^）时，停药后复发风险增加28％。高斜率组（BCR-ABL^IS^显著增加）停药后有87.9％的患者复发，负/低斜率组（BCR-ABL^IS^无显著增加）有27.5％的患者复发。因此，研究建议在12个月剂量减半期后，未丧失MMR并且BCR-ABL^1S^斜率为负/低时的患者才停止治疗，斜率高的患者应该返回全剂量TKI治疗，因为未来极有可能复发。在剂量减少期间的BCR-ABL动力学变化可能有助于在剂量减少期后决定是否停止TKI治疗。

另一项研究对比分析了减少剂量和标准剂量患者停用TKI的结局[35]，研究纳入77例服用一代或二代TKI至少5年、获得DMR至少2年的患者，其中22例患者在停药前至少减量治疗1年，其余55例患者一直使用标准剂量治疗。低剂量组65.4％的患者经历了至少 50％的剂量减少，16例患者接受了二代TKI（61.5％）。分子学复发定义为失去MMR。在停药12个月和60个月时，低剂量组的TFR率高于标准剂量组，分别为80％对56.8％，58.8％对47.5％。停药后复发的多因素分析显示：分子学复发的比例仅与Sokal评分有关，与TKI剂量减少的程度或TKI的类型无关。该研究认为，在停止治疗前至少12个月服用低剂量TKI不会影响TFR的实现，TKI剂量减少的程度、TKI种类不影响分子学复发。

最近一项研究回顾性分析了一代或二代TKI低剂量治疗CML患者的疗效与安全性[36]。研究共298例患者数据可供分析，其中伊马替尼、达沙替尼、尼洛替尼、博苏替尼组患者分别有90、88、81、39例。所有患者至少获得稳定MMR，其中有204例（68.4％）获得MR4。在中位随访27.3个月时，298例患者中有274例（91.9％）维持MMR，减量前MR4持续时间与维持MMR相关，分子学复发定义为失去MMR。达沙替尼组的剂量为70 mg、50 mg、40 mg和≤20 mg，2年无分子学复发生存率分别为100％、96.9％、91.7％和88.5％。值得注意的是，MR4组患者减量后，171例（83.8％）维持或加深了分子学反应水平，MMR组减量后有51例（54.2％）患者分子学反应加深。76例仍维持MR4或更深层次分子学反应的患者停止TKI治疗，21例患者在停药后中位3.6个月失去MMR。所有患者的2年TFR率为74.1％，伊马替尼、达沙替尼、尼洛替尼和博苏替尼组的2年TFR率分别为79.2％、56.3％、76.2％、100％。停药前TKI治疗时间和MR4维持时间与更高的TFR率相关。以上研究结果显示，低剂量TKI的治疗方案也可以使患者有效维持MMR。对于获得MR4以上疗效的患者，减量治疗并不影响TFR的实现。值得注意的是，该研究中停药前TKI维持时间和MR4维持时间与较高的TFR有关。因此，该研究认为，DMR患者的减量可能延长TKI治疗时间和DMR维持时间，增加了TFR的机会。

三、TKI剂量优化的个体化治疗要点及推荐剂量

越来越多的研究证明，为了减少不良反应，提高治疗依从性，同时保证疗效，最终使更多的患者提高生活质量、达到TFR，在治疗的各个阶段对CML-CP患者进行剂量优化的个体化治疗是可行的治疗策略：①已有证据证实，初诊患者优化部分TKI剂量（如达沙替尼50 mg/d）是可行的，如有条件，也可根据血药浓度监测调整剂量。②对于获得MMR以上疗效的患者，降低TKI剂量可在缓解不良反应的同时不丧失疗效甚至继续取得更高疗效。值得注意的是，在调整达沙替尼剂量后，仍有部分患者出现胸腔积液的复发，因此对于已经获得MMR或DMR的患者，在胸腔积液发生前减少剂量可能有助于降低这种特异性不良反应的发生率。需要强调的是，患者在获得MMR或DMR后多长时间可以减量，目前尚无定论。③对于获得稳定DMR的患者，减量似乎不影响其TFR率。此外，在剂量减少期间的BCR-ABL动力学变化可能有助于筛选出有更高概率获得TFR的人群。

基于多项研究的分析结果，《慢性髓性白血病中国诊断与治疗指南（2020年版）》中对不同TKI因不良反应的优化剂量进行推荐[3]，详见表1。

**表1:** 酪氨酸激酶抑制剂初始及调整剂量[3]

剂量选择	治疗剂量
伊马替尼	
首选剂量	400 mg/d
减低剂量	300 mg/d
达沙替尼	
首选剂量	100 mg/d
减低剂量	70 mg/d，50 mg/d
尼洛替尼	
首选剂量	300 mg每日2次，400 mg每日2次（二线治疗）
减低剂量	400 mg，每日1次

四、结论和展望

大多数CML患者需要长期服用TKI，剂量优化不仅可维持疗效，还可以降低不良反应发生率、提高生活质量、降低治疗费用、提高依从性。然而，目前剂量优化的临床数据仍较少，较多问题有待于进一步的大宗临床研究证实，因此建议常规应用于临床，临床医师应在规范化治疗的基础上综合患者病情，如疗效、合并症、不良反应以及治疗意愿等进行个体化的剂量优化选择。
